# Knowledge and attitude toward emergency contraceptive pills among first-year undergraduate students in Southern Thailand

**DOI:** 10.1186/s12909-022-03659-2

**Published:** 2022-08-01

**Authors:** Siranee Yongpraderm, Suriyon Uitrakul, Pakwan Daengnapapornkul, Rawiporn O-in, Bunthita Sinsangbun

**Affiliations:** Department of Pharmaceutical Care, School of Pharmacy, Walailak University, Nakhon Si Thammarat, 80160 Thailand

**Keywords:** Emergency contraceptive pill, Knowledge, Attitude, Undergraduate students

## Abstract

**Background:**

First-year undergraduates are at risk of unexpected pregnancy due to changes in their lives. Adequate knowledge and attitudes towards emergency contraceptive pills (ECPs) are essential to help prevent pregnancy. The objective of this study was therefore to investigate knowledge and attitudes towards ECPs among first-year undergraduate students in a university in Thailand.

**Methods:**

This cross-sectional survey study was performed using developed questionnaires that were validated by four experts. The questionnaires were distributed to all first-year students at the university via an online platform. The characteristic data were descriptively analysed, and the knowledge data were analysed using the chi-square test, Mann‒Whitney U test and one-way ANOVA.

**Results:**

Data from a total of 335 students who responded to the questionnaires and met the eligibility criteria for the study were analysed. The mean knowledge score of all respondents was 7.76 ± 0.15 out of 15. The most correctly answered questions were those relating to the efficacy and safety of ECPs in pregnant women (78.5% and 72.2% correctly answered, respectively). In contrast, the least correctly answered questions were about the ECP regimens and using ECPs instead of combined oral contraception (COC) (30.4% and 34.9%, respectively). In addition, the results indicated that experience in using ECPs and in ECP education were significant factors in high knowledge scores. Moreover, most respondents trusted and would like to receive information on ECPs from health professionals in hospitals, academic institutions, or pharmacies.

**Conclusion:**

The average knowledge of ECPs of first-year students in a university in Thailand was at a moderate level. More information about the regimens of the drugs and the use of ECPs instead of COC should be provided to students, particularly at universities or pharmacies, and should be performed by health care staff.

## Introduction

Emergency contraceptive pills (ECPs) are defined as medicines that can be used to prevent pregnancy after sexual intercourse [1]. There are three regimens of ECPs that are recommended by the World Health Organization (WHO), including ulipristal acetate, levonorgestrel, and combined oral contraceptives (COCs) consisting of ethinyl oestradiol plus levonorgestrel. However, in Thailand, the only ECP regimen registered by the Thai Food and Drug Administration (FDA) is levonorgestrel alone.

According to the information from the International Consortium for Emergency Contraception, several countries allow direct access to ECPs with or without pharmacists. Thailand is one of the countries where ECPs must be dispensed by pharmacists without a doctor’s prescription [2]. Although Thai people must obtain ECPs from pharmacists, many of the studies conducted in Thailand have reported low to moderate levels of knowledge related to ECPs, especially in adolescents and young adults [3–6]. Inappropriate usage of ECPs could result in multiple adverse effects, such as an ectopic pregnancy, vaginal bleeding, and pregnancy complications [7].

Focusing on adolescents and young adults, the first-year undergraduate period should be considered the period in their lives with the most change. In Thailand, before going to universities, students usually live with their parents; however, when enrolling as freshmen at universities, most of them must leave their homes and live in university dormitories. This situation, without or away from parental control, leads to a high risk of sexual intercourse, and adequate knowledge and a positive attitude towards ECPs is one of the methods to prevent unplanned pregnancy [8]. A report from the National Statistical Office of Thailand indicated that of all pregnant women in 2019, approximately 23% had unintended pregnancies [9]. This resulted in determining the occupation of the mothers because almost all of the women with unintended pregnancies were students; being pregnant led the mothers to drop out of their schools or university [10].

According to a survey, one of the causes of unintended pregnancies, especially in teenagers, was the misuse of contraceptives, including ECPs [9]. Several studies reported on the knowledge of ECPs among Thai students in schools and universities [11–13], but none of them focused particularly on first-year undergraduate students in universities. Therefore, this study aimed to investigate the knowledge and attitudes towards ECPs among first-year undergraduate students in a university in Thailand. Additionally, personal factors related to knowledge and attitudes were analysed.

## Materials and methods

### Study population

This cross-sectional descriptive study was conducted at a university in Nakhon Si Thammarat, South Thailand, between 1 December 2019 and 31 January 2020. The inclusion criteria included all first-year undergraduate students who had finished high school or college in Thailand, who were able to read and write in Thai, and who had registered for the first time at the university. Students who did not complete all questions in the survey were excluded. The sample size for this study was calculated using the Krejcie and Morgan formula, resulting in 325 students as the minimum number of respondents.

### Data collection

An online questionnaire was developed. The questionnaire consists of 3 sections. The first section of sociodemographic characteristics includes questions regarding sex, religion, parents’ salary, experience using ECPs and experience receiving education about ECPs. The second section contains 15 questions related to the knowledge of ECPs, including questions regarding indications, contraindications, efficacy, usage, and side effects. The last section measures attitudes regarding ECPs; participants must rate suitable settings for education regarding ECPs and suitable persons for providing education regarding ECPs. In addition, participants are asked whether they prefer using ECPs if needed.

Based on the recommendation by Lynn, a minimum of three experts should be used to review a questionnaire [14]. The questionnaire developed for this study was therefore validated by four experts at the university. The complete online questionnaire was distributed to all first-year undergraduate students at the university for two months. In addition, the researchers advertised this questionnaire by asking lecturers and student leaders to inform all first-year students about the questionnaire before their classes to increase the response rate.

### Statistical analysis

The students’ answers were collected in 3 parts; sociodemographic characteristics and attitudes were collected as nominal numbers, while the scores of correct answers were automatically calculated by the computer. The sociodemographic characteristics of the respondents were descriptively analysed as frequencies and percentages. The scores of knowledge were analysed as the mean and standard deviation (SD). The Mann‒Whitney U test was used to analyse the difference in knowledge among participants with different characteristics. Moreover, the knowledge scores were categorized based on the study of Meechai et al. [11]. Scores of 0–5, 6–10, and 11–15 were defined as low, moderate, and high levels of knowledge, respectively. The characteristics, such as experience using ECPs and receiving education about ECPs, were the independent variables, while the knowledge of the respondents was the dependent variable. Attitudes towards ECPs was described as sequential preference, and one-way ANOVA with post hoc analysis was used for comparing the selected options. Normality tests, including the Kolmogorov‒Smirnov test, were performed to test the appropriateness of the data before one-way ANOVA was performed.

## Results

### Sociodemographic characteristics

A total of 435 students responded to the survey; however, 100 students were excluded because they were not registering at the university for the first time. Therefore, 335 students were analysed in this study. Of these students, 275 students (82.1%) were female, and 289 students (86.3%) were Buddhist (Table 1). The majority of respondents had a monthly salary of 4000–6000 Thai Baht (53.4%), followed by less than 4000 (30.4%) and more than 6000 (16.1%). Most students had never taken ECPs (89.0%) but had been educated about ECPs (74.6%). There were 6 male students (16.2%) who had used ECPs. The sources of ECPs education were academies, self-education and health professionals.

**Table 1:** Characteristics of students responded to the questionnaire (*n* = 335)

Characteristics	Number (%)
Gender	
Male	60 (17.9)
Female	275 (82.1)
Religion	
Buddhism	289 (86.3)
Islam	38 (11.3)
Christianity	2 (0.6)
No religion	6 (1.8)
Monthly salary	
< 4000 Baht	102 (30.4)
4000 – 6000 Baht	179 (53.4)
> 6000 Baht	54 (16.1)
Having experience of using ECPs	
Yes	37 (11.0)
No	298 (89.0)
Having experience of ECPs education	
Yes	250 (74.6)
No	85 (25.4)

### ECPs knowledge scores

Table 2 describes all questions and percentages of the respondents who answered correctly. The question that most students answered correctly was related to the efficacy of ECPs in preventing pregnancy (78.5% answered correctly), followed by the safety of ECPs for pregnant women (72.2% answered correctly) and the efficacy of ECPs in preventing sexually transmitted diseases (66.3% answered correctly). The questions that the fewest students answered correctly were related to the comparison of ECPs and usual contraceptive pills (30.4% answered correctly), the appropriate time to take ECPs (within 72 h post-intercourse) (34.9% answered correctly), and the effect of ECPs in causing abortion (36.7% answered correctly).

**Table 2:** Percentages of respondents who correctly answered the questions of knowledge about emergency contraceptive pills (ECPs) (*n* = 335)

Question	Number (%)
Can ECPs treat pimples?	131 (39.1)
Can ECPs prevent sexually transmitted diseases?	222 (66.3)
Can ECPs be used as usual contraception (e.g. combined oral contraceptive pills; COCs)?	156 (46.6)
Is the most effective time for taking ECPs before sexual intercourse?	216 (64.5)
Should the second tablet of ECPs be taken 12 h after the first tablet?	169 (50.4)
Should ECPs be taken within 72 h after sexual intercourse?	117 (34.9)
Can ECPs completely (100%) prevent pregnancy?	263 (78.5)
Are ECPs more effective to prevent pregnancy than COCs?	102 (30.4)
Is the most effective time for taking ECPs within 12 h after sexual intercourse?	207 (61.8)
Are nausea and vomiting side effects of ECPs?	217 (64.8)
Is vaginal bleeding a side effect of ECPs?	136 (40.6)
Is ectopic pregnancy a side effect of ECPs?	141 (42.1)
Is ECPs safe in pregnant women?	242 (72.2)
Can ECPs be taken more than 4 tablets a month?	160 (47.8)
Can ECPs cause abortion?	123 (36.7)

**Fig. 1:**
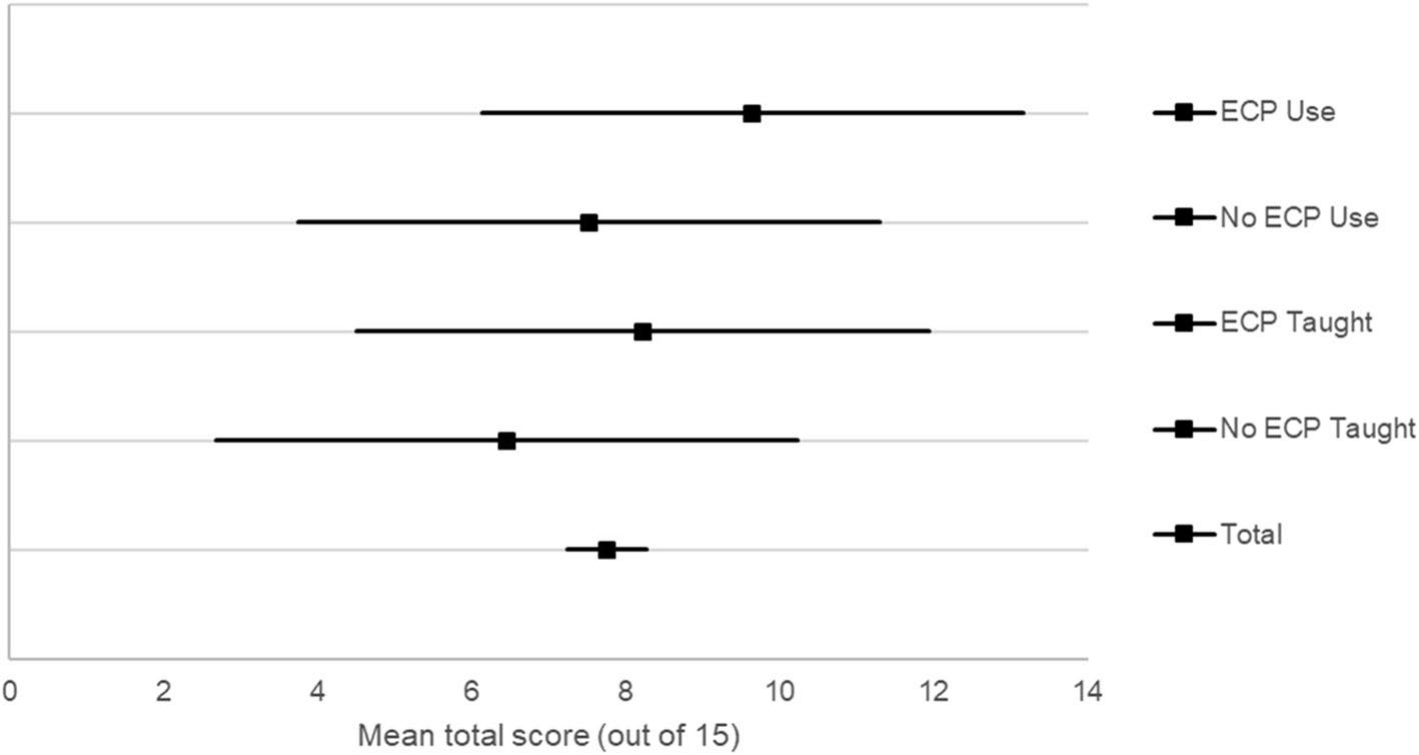
The average total scores and 95% confidence intervals of the respondents with different factors, including had experience in ECP use and had been taught about ECPs

The mean ECP knowledge score of all respondents was 7.76 (95% CI 7.25–8.27) out of 15 (47.5%), which was a moderate level of knowledge (Fig. 1). The median overall score was 7 (interquartile range [IQR] 4–10). The respondents who had experience using ECPs had significantly higher scores than those who had no such experience (9.65 vs. 7.53, *p* value 0.001). The respondents who had been taught about ECPs had significantly higher scores than those who had not been taught (8.23 vs. 6.46, *p* value < 0.001). The respondents with both factors had an average score of 10.31 (95% CI 7.1–13.52), which was significantly higher than the respondents with none of the factors (mean 6.38, 95% CI 2.60–10.16, *p* value < 0.001). Moreover, male participants had insignificantly lower scores than females (6.97 vs. 7.94, *p* value 0.072).

### Preferences in ECPs education

Tables 3 and 4 describe the preferences of the respondents for suitable locations for receiving ECPs education and suitable persons for providing ECPs education, respectively. For location, most students preferred to receive ECPs education at hospitals, academic institutions, and pharmacies rather than online media or webpages. Normality test results showed that the *p* values of all choices of location were more than 0.05, so one-way ANOVA was used to analyse the mean preference scores. The results indicated no significant difference in mean preference scores among hospitals, academic institutions, and pharmacies (*p* values > 0.05, Table 3). On the other hand, these scores were significantly higher than the mean preference scores for online media or webpages (*p* values < 0.05, Table 3).

**Table 3:** Differences in average preference scores of suitable locations for education of emergency contraceptive pills (max score = 5)

	Academic institution Mean = 3.20	Pharmacy store Mean = 3.09	Online media Mean = 2.78	Webpage Mean = 2.69
Hospital Mean = 3.25	0.05 (*p* = 0.66)	0.16 (*p* = 0.14)	0.47 (*p* < 0.001)	0.56 (*p* < 0.001)
Academic institution Mean = 3.20		0.11 (*p* = 0.29)	0.42 (*p* < 0.001)	0.51 (*p* < 0.001)
Pharmacy store Mean = 3.09			0.31 (*p* = 0.01)	0.40 (p < 0.001)
Online media Mean = 2.78				0.09 (*p* = 0.42)

One-way ANOVA with post-hoc analysis of Tukey was performed. Sample size was 335

**Table 4:** Differences in average preference scores of suitable persons for education of emergency contraceptive pills (max score = 5)

	Parents Mean = 3.26	Teachers Mean = 3.04	Friends Mean = 2.78	Lovers Mean = 2.69
Health professionals Mean = 3.56	0.30 (*p* = 0.01)	0.52 (*p* < 0.001)	0.78 (*p* < 0.001)	0.87 (*p* < 0.001)
Parents Mean = 3.26		0.22 (*p* = 0.04)	0.48 (*p* < 0.001)	0.57 (*p* < 0.001)
Teachers Mean = 3.04			0.26 (*p* < 0.001)	0.35 (*p* < 0.001)
Friends Mean = 2.78				0.09 (*p* = 0.13)

One-way ANOVA with post-hoc analysis of Tukey was performed. Sample size was 335

For suitable persons, most students preferred receiving knowledge from health professionals, parents, and teachers rather than their friends or their lovers. Statistical analysis showed significantly higher mean preference scores for health professionals than parents (*p* value 0.01) and for parents than teachers (*p* value 0.04) (Table 4). However, the mean preference scores for friends or lovers were not significantly different (*p* value 0.13, Table 4).

For reasons to use ECPs, the first reason was the efficacy in preventing unintended pregnancy (mean score 3.29, 95% CI 1.83–6.58). The convenience of getting ECPs and simple instructions were also important reasons that led the respondents to use ECPs (mean scores 3.17 (95% CI 1.76–4.58) and 3.08 (95% CI 1.98–4.18), respectively). Safety and price were the least important factors for using ECPs among all respondents, with mean preference scores of 2.75 (95% CI 1.39–4.11) and 2.54 (95% CI 0.95–4.13), respectively. However, the mean score of the reason for efficacy was not significantly different from that for convenience (*p* value 0.244), while the mean score for convenience was not significantly different from that for simple instruction (*p* value 0.421). Moreover, 86.0% of the participating students said that they were going to use ECPs in case of unprotected sex.

## Discussion

The results from this survey study indicated that first-year undergraduate students at a university in southern Thailand had a moderate level of knowledge about emergency contraceptive pills (ECPs), with an average score of 7.76 out of 15 (51.73%). The greatest preference of the students for places to receive education about ECPs was hospitals, and that for the proper persons to provide education about ECPs were health professionals. In addition, the most important reason to use ECPs was the efficacy in preventing unintended pregnancy.

Knowledge and attitudes towards ECPs are an interesting topic to study globally, especially among adolescents and young adults. For example, there have been several studies in the United States [15, 16], India [17], Korea [18], Cameroon [19], Nigeria [19], and Thailand [3, 4]. The results from almost all studies indicated that college students and undergraduate students had a poor to moderate level of knowledge about ECPs, although most of them had positive attitudes towards the drugs. This phenomenon highlighted that the lack of knowledge regarding ECPs is a problem worldwide and seems not to be related to the area of study. Students from a developed country, i.e., the United States, had similar knowledge to those from developing countries, e.g., Thailand and Cameroon.

Focusing on Thailand, a study by Aimnoi et al. revealed that the majority (58.2%) of undergraduate students in Bangkok had a low level of knowledge, while 32.1% had a moderate level of knowledge [3]. In addition, 67.9% of the students knew the indications of the drugs, but 39.3% of them provided wrong answers regarding drug regimens. Most students (71.1%) were also unaware of the side effects. These results indicated the consistency of knowledge regarding the location of the population. Students who live in Bangkok had better opportunity to receive more knowledge than students in the current study, which was performed in a rural area of Thailand. However, the abovementioned results indicated that easier access to health care professionals was not related to better knowledge regarding ECPs. Another study was performed with 210 university students outside Bangkok, and it showed similar results [4]. Over 80% of the students knew the indications of ECPs; however, approximately 36% of the students had misconceptions about the side effects of the drugs. These results were in concordance with this study; almost 80% of all respondents knew that ECPs had efficacy in preventing unintended pregnancy, while only approximately 30% knew the side effects of abortion and correct regimens of the drugs. Although the study by Bennhult Hansson et al. did not mention the exact location of the participants [4], the results highlighted a similar situation to the study in Bangkok, as well as the current study.

Although many previous studies have focused on young adults, to the best of our knowledge, none of them have been particularly conducted with first-year undergraduate students. Indeed, college or high school students might be younger than first-year undergraduate students and probably need more attention. This study, however, emphasized first-year undergraduate students due to the environmental change that they experience. In Thailand, most college and high school students stay with their parents or guardians, such as their uncles, aunts, or grandparents, so the possibility of sexual intercourse is fairly low. These students usually move to dormitories when enrolling as first-year undergraduate students and then have a much higher chance of a sexual relationship [20, 21]. Nonetheless, the results in this study showed no difference from previous studies that were performed with college students and undergraduate students.

Regarding the place for receiving ECPs education, most respondents preferred hospitals, academic institutions, and pharmacies. Moreover, they preferred receiving knowledge from health professionals rather than from their teachers or parents. These results indicated that students were confident if the knowledge was imparted at reliable places and from reliable persons. In agreement with the study by Aimnoi et al., the preferred source of information regarding ECPs was qualified medical staff, although students actually received the information from their friends or the internet [3]. However, it should be noted that, based on the results of students in Bangkok [3], a higher number of hospitals and pharmacies in Bangkok did not result in higher knowledge of the students. This issue should be further discussed to determine why undergraduate students did not obtain knowledge from their nearby health care staff. One possible reason could be that asking health care professionals makes students too shy. Some studies, therefore, have suggested that contraceptive knowledge should be conveniently accessible, such as at home or school [22].

Several limitations of this study need to be discussed. First, the developed online questionnaire was not tested for reliability or consistency using a pilot study due to the limitations of time and available students. However, the questionnaire was reviewed by four experts from four different schools in the university, so the content should be adequately reliable. Later, because this study used online questionnaires, respondents were unable to contact the researchers if they were unclear about the questions. For instance, there were some male respondents who answered “Have ever used ECPs”; it was unsure that they understood the word “use”. It is possible that some male students who bought ECPs for their girlfriends misunderstood the questions and thought this was the “use of ECPs”. Last, only a few personal characteristics of the respondents were collected in this study. Neither the course of study nor the accommodation type of the respondents were collected due to the recommendation from the ethics committee. Students in health science courses such as medicine, pharmacy, and nursing might have more knowledge than non-health science students. Moreover, students who stay in private accommodations might have a greater chance of having sex and might have different knowledge than those who stay in university dormitories. These factors should be considered and collected in future research.

## Conclusion

This cross-sectional survey study was performed with first-year undergraduate students at a university in southern Thailand. The results showed that the respondents had moderate knowledge of emergency contraceptive pills. The level of knowledge related to the indications and efficacy of the drugs was the highest, while that related to side effects and using regimens of the drugs was the lowest. Moreover, the preferred source of ECPs information was from health professionals, and the preferred locations were hospitals, academic institutions and pharmacies.

## Data Availability

The datasets used and analysed during the current study are available from the corresponding author on reasonable request.
